# CBT for depression and anxiety adapted for psychosis risk in primary care: controlled trial to assess feasibility, acceptability and signals of efficacy

**DOI:** 10.1192/bjo.2025.27

**Published:** 2025-05-15

**Authors:** Katherine Newman-Taylor, Tess Maguire, Tanya Smart, Emma Bayford, Emily Gosden, Grace Addyman, Jessica Grange, Pete Bullard, Miriam Simmons-Dauvin, Morad Margoum, Ben Smart, Keith Das, Sophie Hardy, Catherine Hiscutt, Charlotte Hodges, Adam Holleyman, Hettie Jones, Kate Spurr, Jessica Trickett, Elizabeth Graves

**Affiliations:** School of Psychology, University of Southampton, Southampton, UK; Psychology, Southern Health NHS Foundation Trust, Southampton, UK; Talking Therapies, Solent NHS Trust, Portsmouth, UK; Talking Therapies, Isle of Wight NHS Trust, Newport, UK; Talking Therapies, Dorset HealthCare University NHS Foundation Trust, Bournemouth, UK

**Keywords:** CHR-P, ARMS, early intervention, Talking Therapies, attachment

## Abstract

**Background:**

People at high risk for psychosis access primary care mental health services for depression and anxiety and are unlikely to recover from these affective symptoms. We report the first controlled trial of cognitive–behavioural therapy (CBT) for depression and anxiety, minimally adapted for psychosis risk, in primary care.

**Aims:**

To evaluate feasibility, acceptability and signals of efficacy for CBT for depression and anxiety adapted for psychosis risk, designed in collaboration with people with psychosis.

**Method:**

A longitudinal controlled trial comparing best practice CBT for depression and anxiety (CBT-BP) with CBT adapted for psychosis risk (CBT-PR), in patients meeting criteria for UK primary care services and who are also clinically high risk for psychosis (trial registration no. ISRCTN40678).

**Results:**

Rates of recruitment (55 to CBT-BP, 44 to CBT-PR), completion of measures (90% CBT-BP, 94% CBT-PR) and retention in therapy (75% CBT-BP, 95% CBT-PR) demonstrate the feasibility and acceptability of the adapted therapy. Routine measures of depression and anxiety signal improved clinical and recovery outcomes for CBT-PR. Psychosis and relational measures signal sustained improvement (at 3 months) in the CBT-PR group. No serious adverse events were reported.

**Conclusions:**

Primary care mental health services present a unique opportunity to identify and treat people at risk of psychosis at a time when they are help-seeking. CBT for depression and anxiety, minimally adapted for psychosis risk, can be delivered in routine services, and is likely to improve clinical and recovery outcomes and reduce psychosis risk. A definitive trial is needed to estimate clinical and cost-effectiveness.

Primary care mental health services for depression and anxiety are routinely accessed by people with concurrent psychotic symptoms, such as hearing voices and paranoia.^1–3^ Attenuated psychotic symptoms, short-lived and remitting psychotic symptoms, and/or decreased functioning in the context of familial risk, indicate clinical high risk for psychosis (CHR-P).^4^ A third of this group transitions to psychosis within 3 years of initial presentation.^5,6^ Despite evidence of CHR-P in people seeking help for depression and anxiety, primary care services do not routinely screen for psychosis risk.^7^

Early psychological interventions reduce transition to psychosis by 45% at 12 months, and by 40% at 18–48 months, of follow-up^a^ and are likely to be cost-effective and cost-saving.^9^ UK and US psychiatric morbidity surveys show that people at CHR-P are twice as likely to seek help, and typically do so for affective presentations.^10,11^ Primary care services that deliver evidence-based psychological interventions for depression and anxiety are therefore well placed to detect and treat psychosis risk.^3^

## A recovery gap in clinical outcomes for affective presentations

In UK primary care mental health services, people at CHR-P report higher levels of depression and anxiety at assessment and are less likely to have recovered by the end of treatment (27%) compared with those with no psychosis risk (62%).^1^ The failure to identify CHR-P and close the recovery gap in clinical outcomes for people with and without psychosis risk comes at significant human and financial cost. A recent study, utilising a two-item screen constructed from Bayesian analyses of previously validated tools,^12^ demonstrated that a very brief measure of CHR-P incorporated in primary care triage assessments predicted severity of depression and anxiety, mental and physical comorbidities and poorer recovery trajectories.^2^ No studies have yet examined the feasibility, acceptability and potential impact of augmented psychological therapies for this population in primary care settings.

In the UK, the great majority (∼90%) of adults with mental health problems are treated in primary care through the National Health Service (NHS).^13,14^ Within the NHS, Talking Therapies^b^ services deliver evidence-based psychological therapies for depression and anxiety presentations,^14^ with major effects on measures of both.^15^ These services were not designed for people with CHR-P but are routinely accessed by individuals with greater levels of severity and complexity than were originally intended.^16^ Psychosis risk typically goes undetected, and the affective presentations for which people are seeking help are treated suboptimally.^1–3^

Primary care mental health services present a scalable opportunity to deliver very early interventions to people at risk of psychosis at a time when they are seeking help, and via services designed and equipped to deliver evidence-based therapies targeting the problems for which they are seeking treatment. This opportunity for early intervention contrasts with well-documented delays to treatment for established psychosis of typically 12–24 months, with attendant personal, societal and health economic costs.^17,18^

## Current study

In line with the Medical Research Council framework for developing and evaluating complex interventions,^19^ we worked closely with key stakeholders (including people with lived experience of psychosis risk and accessing primary care mental health services, family members and clinicians) to develop minimal adaptations to cognitive–behavioural therapy (CBT) for the implementation of depression and/or anxiety in these settings. Adaptations were based on evidence that people at psychosis risk first access primary care for affective presentations^10,11^ and are treated suboptimally.^1–3^ The purpose of the current study was to assess the feasibility and acceptability of the intervention prior to progression to a definitive trial.^19^

This is the first study to (a) assess the feasibility and acceptability of minimally adapted CBT for depression and/or anxiety, taking account of CHR-P, in routine primary care mental health services; (b) examine signals of efficacy compared with routinely delivered evidence-based CBT for depression and/or anxiety; and (c) explore the role of probable indicators of complexity – sociodemographic, clinical and relational factors – on therapeutic engagement and outcomes.

## Method

The authors assert that all procedures contributing to this work comply with the ethical standards of the relevant national and institutional committees on human experimentation, and with the Helsinki Declaration of 1975 as revised in 2013. All procedures involving human subjects/patients were approved by the UK NHS Research Ethics Committee and Health Research Authority (ID no. 21/ne/0206) and the UK University of Southampton (ID no. 64425), and were preregistered with the Open Science Framework registry (ID no. osf.io/vd87x). The study aligns with the Consolidated Standards of Reporting Trials (CONSORT) extension for pilot and feasibility trials^20^ (see supplementary material, CONSORT checklist).

### Patient and public involvement

In order to improve the quality and impact of the research, a patient and public involvement (PPI) group was formed for the study, consisting of people with direct or indirect experience (through family members) of CHR-P and accessing NHS Talking Therapies. The research team met with the group over the course of the project to discuss study design, recruitment and implementation.

### Design

We used a longitudinal, two-arm, non-randomised controlled design to examine the feasibility, acceptability and potential efficacy of minimally adapted CBT for patients meeting criteria for UK primary care services for depression and/or anxiety and who are also CHR-P. To assess signals of efficacy, we compared outcomes for the adapted CBT taking account of psychosis risk (CBT-PR) from 1 April 2022 to 31 March 2024 (intervention and follow-up period) with controls who received best practice CBT for depression/anxiety^c^ (CBT-BP) from 1 October 2021 to 31 March 2022 (control period).^d^

### Setting and sample

Three NHS Trusts participated in the study, serving a combined population of ∼670 000 people, across city, urban and rural locations in the UK. Embedded Talking Therapies services deliver evidence-based CBT and other psychological interventions for depression and anxiety presentations to adults aged 16 years and above.^14^ People may either self-refer or be referred by other healthcare clinicians.

**Fig. 1:**
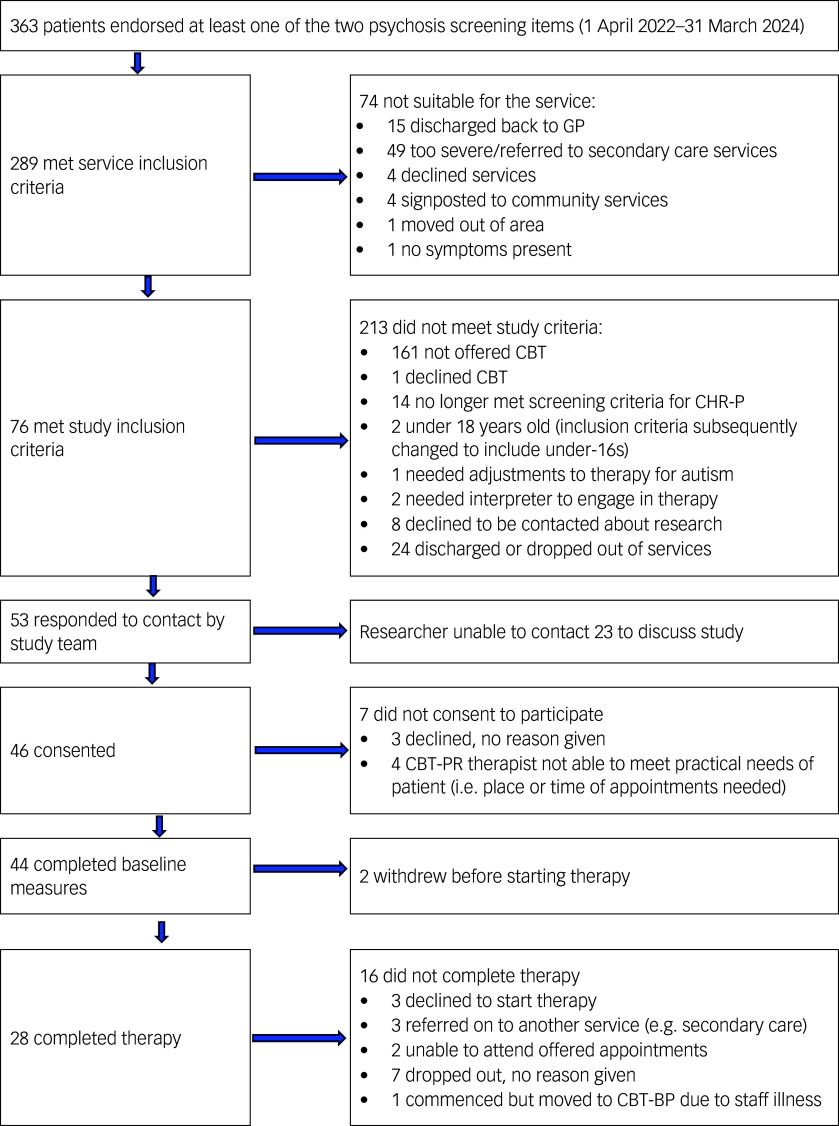
CONSORT diagram. CONSORT, Consolidated Standards of Reporting Trials; GP, general practitioner; CBT, cognitive–behavioural therapy; CHR-P, clinical high risk for psychosis; CBT-PR, CBT adapted for psychosis risk; CBT-BP, best practice CBT.

Individuals who accessed participating services between 1 April 2022 and 31 March 2023, met service criteria (primary diagnosis of depression and/or anxiety) and were identified as CHR-P (using a two-item screen; see Measures, below) were invited to participate. A total of 44 participants consented and received CBT-PR. The control group consisted of those 55 participants who were identified as CHR-P and received CBT-BP (as outlined above) during the control period (1 October 2021–31 March 2022). The full sample was aged between 17 and 62 years (*M* = 28, s.d. = 11.55), of which the majority were female (58.2% women, 40% men, 1.8% non-binary). See Fig. 1 for CONSORT diagram, and supplementary material (S1) for full demographic details.

### CBT for depression and/or anxiety, minimally adapted for psychosis risk

Best practice CBT protocols for depression and anxiety presentations, identified by systematic reviews and meta-analyses of the empirical literature,^21^ and typically supported by National Institute for Health and Care Excellence guidelines, are delivered by NHS Talking Therapies.^14^ These protocols guide clinicians to (a) develop an individualised ‘formulation’ – a diagrammatic representation of cognitive, behavioural and affective factors contributing to the development and maintenance of distress; (b) support reflection on the impact of key cognitive (e.g. self-critical appraisals) and behavioural (e.g. avoidance) responses to triggers; and (c) encourage development of alternative responses likely to reduce distress and improve quality of life (e.g. re-evaluating self-critical appraisals, or gradually facing feared situations to build self-efficacy even when anxious) if the person so chooses.

Trial clinicians received a 3.5 h online training session on (a) what constitutes psychosis risk, (b) prevalence of psychosis risk in people accessing primary care mental health services for depression and/or anxiety and (c) adaptations to therapy as usual for this group. Minimal adaptations to CBT for the current study included (a) measuring psychosis risk and relational (attachment) factors using brief validated self-report tools, (b) naming psychosis and relational factors in the formulation and (c) following original protocols for depression and/or anxiety.

Following consultation with people with lived experience of psychosis risk and of accessing these services, clinicians asked about psychosis experiences in a warm, non-judgemental and destigmatising manner (e.g. ‘Many people see or hear things that others don’t see or hear, especially when under stress. You’ve said [indicating the questionnaire] that you sometimes hear whispering – when does this tend to happen, and how does this leave you feeling?’). This can then be linked to the formulation of their depression and/or anxiety (e.g. ‘So, along with thinking about leaving the house or seeing other people, the whispering can trigger intense feelings of anxiety, and you cope by staying in alone. This helps briefly but the next time you think about going out, the whispering and anxiety come back strongly – have I understood this correctly?’). These psychosis experiences are then simply named in the formulation of the person’s depression and/or anxiety (e.g. whispering as an additional trigger for a typical cycle of anxiety and accompanying avoidance). The clinician then follows the protocol for depression and/or anxiety presentation as usual.

### Procedure

People accessing UK primary care services, and who met the usual service criteria for CBT for depression and/or anxiety from 1 October 2021 to 31 March 2022 (control period) and endorsed at least one of the two items of the CHR-P screen,^12^ received treatment as usual. This included best practice CBT (see University College London Competence Framework: http://www.ucl.ac.uk/pals/research/cehp/research-groups/core/competence-frameworks), alongside assessment of sociodemographic data (age, ethnicity, gender, relationship and employment status, postcode, religious group and any disability or long-term condition), presenting problem, nationally mandated patient-reported outcome measures for depression and anxiety (pre- and post-intervention) and a two-item CHR-P screen.^12^

People accessing the same services, who met the usual service criteria for CBT for depression and/or anxiety from 1 April 2022 to 31 March 2023 (intervention recruitment period) and endorsed at least one of the two items of the CHR-P screen^12^ were invited to participate in the study. Participants gave informed written consent and then completed the additional measure of CHR-P and three relational measures (see Measures, below). They then commenced the adapted CBT (CBT-PR). Standard service data regarding sociodemographic information, presenting problem and nationally mandated outcome measures of affective presentations were gathered as described above.

Therapists for both intervention and control groups were CBT trained and British Association for Behavioural and Cognitive Psychotherapy (BABCP)^e^-accredited practitioners. All data were gathered by the clinical team and entered into the service database, in line with routine practice. Pseudonymised data were passed to the research team following removal of personally identifiable information.

### Measures

#### Psychosis risk

Two-item screens for early psychosis were constructed from Bayesian analyses of previously validated screening measures.^12^ We used the Abbreviated Youth Psychosis at Risk Questionnaire items 12 and 22 to optimise specificity (73%) and sensitivity (65%) in help-seeking populations.

The Prodromal Questionnaire-16 (PQ-16)^22^ is a 16-item measure of CHR-P. This measure assesses the presence of attenuated psychotic symptoms (yes/no), and distress associated with items endorsed, using a four-point Likert scale (from ‘no’ to ‘severe’). Responses are summed for distress scores (0–48) or total number of symptoms endorsed (0–16). Internal consistency is acceptable (*α* = 0.77).

#### Depression, anxiety and functioning

The Patient Health Questionnaire-9 (PHQ-9)^23^ is a nine-item measure of depression over the previous 2 weeks. Items are rated on a four-point Likert scale (from ‘not at all’ to ‘nearly every day’). Responses are summed, with higher scores reflecting greater levels of depressed mood. Internal consistency is good (*α* = 0.89).

Generalised Anxiety Disorder-7 (GAD-7)^24^ is a seven-item measure of generalised anxiety over the previous 2 weeks. Items are rated on a four-point Likert scale (from ‘not at all’ to ‘nearly every day’). Responses are summed, with higher scores reflecting greater levels of anxiety. Internal consistency is excellent (*α* = 0.92).

The Work and Social Adjustment Scale (WSAS)^25^ is a five-item measure of impaired functioning. Items are rated on a nine-point Likert scale (from ‘not at all’ to ‘very severely’). Responses are summed, with higher scores reflecting greater levels of impairment. Internal consistency is acceptable to excellent (*α*s > 0.70).

#### Relational measures

The Psychosis Attachment Measure-Revised (PAM-R)^26^ is a 26-item self-report measure of adult attachment. Items are rated on a four-point Likert scale (from ‘not at all’ to ‘very much’). Responses are summed, with higher scores reflecting greater levels of attachment anxiety, avoidance or disorganisation. Internal consistency is good for anxiety (*α* = 0.82) and acceptable for both avoidance (*α* = 0.76) and disorganisation (*α* = 0.76) subscales.

The Experiences in Close Relationships-Short (ECR-12)^27^ is a 12-item measure of adult attachment. Items are rated on a seven-point scale (from ‘disagree strongly’ to ‘agree strongly’). Responses are summed, with greater scores reflecting higher levels of attachment anxiety or avoidance. Internal consistency is good for anxiety (*α* = 0.81) and acceptable for avoidance (*α* = 0.79) subscales.

The Dysfunctional Working Models Scale (adapted) (DWMS(A), following Perris et al^28^) is a 35-item measure of conditional interpersonal beliefs (underlying assumptions) about self and others. Items are rated on a seven-point scale (from ‘I totally agree’ to ‘I totally disagree’). We adapted the original to include only the 32 items that loaded in the standardisation factor analysis, plus three items designed to represent secure, anxious and avoidant interpersonal beliefs, respectively. Internal consistency for the original scale is excellent (*α* = 0.97), and this was good in the current sample for the adapted scale for both 32-item (*α* = 0.89) and 35-item versions (*α* = 0.89).

### Analysis plan

#### Description of the sample

We summarised sociodemographic, presenting problem and initial clinical measures for the two groups. Group comparisons used *t*-tests for continuous variables, and chi-square for categorical data.

#### Is CBT-PR feasible and acceptable in primary care mental health settings?^f^

To assess feasibility, we calculated (a) rates of recruitment to the study (percentage of patients who consented versus all approached to participate) and (b) completion rates for measures (percentage CBT-BP versus CBT-PR for those who completed all measures). To assess acceptability, we calculated therapy completion rates: (a) percentage CBT-BP versus CBT-PR for those who completed therapy^g^ and (b) number of sessions attended and cancelled for CBT-BP versus CBT-PR.

#### Does CBT-PE signal improvements in clinical and recovery outcomes?

To assess signals of efficacy, we compared first and final scores for depression, anxiety and functioning for all who completed CBT-BP versus CBT-PR.

#### Does CBT-PR signal change in psychosis and relational measures at 3-, 6- and 12-month follow-up?

To assess potential impact on psychosis and relational measures, we calculated changes from pre-therapy to 3-, 6- and 12-month follow-up in the CBT-PR group.

#### Are sociodemographic, clinical and relational factors associated with therapy engagement and outcomes?

To explore the role of probable indicators of complexity, we calculated associations between sociodemographic, clinical and relational factors and therapeutic engagement (sessions attended/cancelled, therapy completion rates) and outcome measures.

## Results

### Sociodemographic characteristics and initial group comparisons

There were no differences between groups in self-reported age, gender, ethnicity or long-term conditions. Similarly, there were no differences in initial measures of depression (PHQ-9), anxiety (GAD-7) and functioning (WSAS), or in the number of sessions not attended.

We did find differences in rates of employment (*X* ^*2*^(5, *n* = 99) = 12.17, *p* = 0.03; CBT-BP, *n* = 13; CBT-PR, *n* = 22), presenting problem (*X* ^*2*^(4, *n* = 99) = 13.00, *p* = 0.01), sessions attended (*t* = −1.94, *p* = 0.05; CBT-BP, *M* = 9.44; CBT-PR, *M* = 12.14) and sessions cancelled (*t* = −3.37, *p* = 0.001; CBT-BP, *M* = 1.36; CBT-PR, *M* = 2.64). See supplementary material for full sociodemographic details (S1) and baseline group comparisons (S2).

### Feasibility and acceptability of CBT-PR in primary care mental health settings

No serious adverse events were reported over the course of the study.

#### Rates of recruitment into the study

Figure 1 shows that 76 patients were screened as being eligible to take part in the intervention, of whom 53 (69.7%) responded to contact from a researcher. Of those who discussed the study with a researcher, 87% consented to participate.

#### Completion rates for measures and therapy

Table 1 gives completion rates for measures and therapy, and indicates high levels of each for both CBT-BP and CBT-PR.

**Table 1:** Feasibility and acceptability outcomes

Measure/variable	Missing, *N* (%)		Completed, *N* (%)	
	CBT-BP	CBT-PR	CBT-BP	CBT-PR
Measures completed (first and last)				
PHQ-9	5 (9)	2 (5)	50 (95)	42 (98)
GAD-7	8 (15)	3 (7)	47 (85)	41 (93)
WSAS	5 (9)	4 (9)	50 (91)	40 (91)
Mean % completed			90	94
Attended 2+ sessions			47 (85)	43 (98)

	*N*		*M* (s.d.)		*t*	*p*	Cohen’s *d*
Measure/variable	CBT-BP	CBT-PR	CBT-BP	CBT-PR			
Sessions attended	55	44	9.44 (7.41)	12.14 (6.12)	−1.94	0.03*	−0.39
Sessions not attended	55	44	1.11 (1.29)	0.93 (1.35)	0.66	0.25	0.14
Sessions cancelled	55	44	1.36 (1.80)	2.64 (2.00)	−3.37	<0.001**	−0.68

PHQ-9, Patient Health Questionnaire-9 measure of depression; GAD-7, Generalised Anxiety Disorder-7 measure of anxiety; WSAS, Work and Social Adjustment Scale measure of functioning; CBT-BP, best practice cognitive behaviour therapy; CBT-PR, cognitive behaviour therapy adapted for psychosis risk. *p < 0.05, **p < 0.001.

#### Sessions attended and cancelled

Table 1 also shows that participants engaged in CBT-PR both attended and cancelled more sessions compared with the CBT-BP group.

### Clinical and recovery outcomes

Table 2 gives clinical and recovery outcomes for depression (PHQ-9), anxiety (GAD-7) and functioning (WSAS), and indicates improved outcomes in measures of both depression and anxiety in CBT-PR compared with CBT-BP.

**Table 2:** Signals of improvement in clinical and recovery outcomes

Measure/variable	*N*		*M* (s.d.)		*t*	*p*	Cohen’s *d*
	CBT-BP	CBT-PR	CBT-BP	CBT-PR			
PHQ 9 first	55	43	19.15 (4.68)	18.56 (5.48)	0.57	0.29	0.12
PHQ 9 last	50	42	15.90 (6.99)	12.71 (7.29)	2.14	0.02*	0.45
GAD 7 first	55	43	15.25 (3.95)	15.26 (3.95)	−0.002	0.50	0.00
GAD 7 last	47	41	13.20 (5.36)	10.49 (5.75)	2.28	0.01*	0.49
WSAS first	55	43	23.76 (9.29)	23.98 (8.36)	−0.12	0.45	−0.02
WSAS last	50	40	21.06 (9.95)	19.65 (9.74)	0.67	0.25	0.14

Measure/variable		*N*		%		*X* ^2^ (d.f.)	*p*
		CBT-BP	CBT-PR	CBT-BP	CBT-PR		
PHQ-9 reliable improvement	Yes	21	22	38	50	1.39 (1)	0.24
	No	34	22	62	50		
GAD-7 reliable improvement	Yes	24	25	44	57	1.70 (1)	0.19
	No	31	19	56	43		
PHQ-9 clinical change	Improvement	15	16	27	36	0.94 (1)	0.33
	No change	40	28	73	64		
GAD-7 clinical change	Improvement	14	22	25	50	6.36 (1)	0.01*
	No change	41	22	75	50		
PHQ-9 reliable recovery	Yes	13	14	24	32	0.83 (1)	0.36
	No	42	30	76	68		
GAD-7 reliable recovery	Yes	13	20	24	45	5.24 (1)	0.02*
	No	42	24	76	55		

PHQ-9, Patient Health Questionnaire-9 measure of depression; GAD-7, Generalised Anxiety Disorder-7 measure of anxiety; WSAS, Work and Social Adjustment Scale measure of functioning; CBT-BP, best practice cognitive behaviour therapy; CBT-PR, cognitive behaviour therapy adapted for psychosis risk. *p < 0.05.

In line with standard national reporting of recovery indices, we compared (a) reliable improvement (indicative of clinically significant change, specified for each outcome measure), (b) clinical change (indicative of moving from above to below probable caseness for depression or anxiety) and (c) reliable recovery (meeting criteria for both reliable improvement and clinical change) for the two groups. Table 2 shows that CBT-PR was associated with greater clinical change and reliable recovery in anxiety (GAD-7) compared with CBT-BP.

### Change in psychosis risk and relational measures at 3- 6- and 12-month follow-up

CBT-PR follow-up data indicate improvements in psychosis risk (PQ-16) and relational measures (PAM-R, ECR-12 and DWM-S(A)) from pre-therapy to 3-, 6- and 12-month follow-up (see Table 3).

**Table 3:** Signals of improvement in psychosis risk and relational measures at follow-up

Measure	*N*	Baseline *M* (s.d.)	3 months *M* (s.d.)	*t*	*p*	Cohen’s *d*	*N*	Baseline *M* (s.d.)	6 months *M* (s.d.)	*t*	*p*	Cohen’s *d*	N	Baseline *M* (s.d.)	12 months *M* (s.d.)	*t*	*p*	Cohen’s *d*
PQ-16	36	22.86 (8.35)	19.94 (9.58)	2.27	0.02*	0.38	30	21.97 (8.19)	17.63 (10.01)	2.78	0.009*	0.51	22	20.95 (9.260)	15.14 (9.75)	3.74	0.001**	0.80
PAM-R disorganised	36	1.79 (0.56)	1.52 (0.68)	2.58	0.014*	0.43	30	1.69 (0.67)	1.43 (0.55)	2.33	0.027*	0.43	22	1.75 (0.590)	1.46 (0.69)	2.66	0.02*	0.77
PAM-R avoidance	34	2.06 (0.55)	1.94 (0.60)	1.63	0.112	0.28	29	2.07 (0.55)	1.92 (0.73)	1.24	0.23	0.23	21	2.33 (0.360)	1.98 (0.68)	2.55	0.02*	0.56
PAM-R anxious	36	1.02 (0.65)	1.74 (0.70)	2.32	0.013*	0.39	30	1.94 (0.67)	1.64 (0.67)	2.74	0.01*	0.50	22	1.78 (0.680)	1.44 (0.63)	2.57	0.02*	0.55
ECR-12 avoidance	35	4.49 (1.16)	4.60 (1.27)	−0.64	0.529	−0.11	30	4.64 (1.10)	4.53 (1.42)	0.43	0.67	0.08	22	5.09 (0.830)	4.54 (1.34)	2.15	0.04*	0.45
ECR-12 anxiety	36	5.42 (1.42)	4.96 (1.72)	2.02	0.052	0.34	30	5.20 (1.45)	4.70 (1.64)	2.08	0.05*	0.38	22	5.04 (1.570)	4.56 (1.48)	2.05	0.053	0.44
DWM-S(A)	35	114.80 (33.92)	119.97 (36.58)	−1.15	0.257	−0.20	28	117.04 (6.99)	130.36 (6.14)	−2.11	0.05*	−0.40	22	114.36 (34.40)	122.50 (49.03)	−0.66	0.51	−0.14

PQ-16, Prodromal Questionnaire-16 measure of clinical high risk for psychosis (CHR-P); PAM-R, Psychosis Attachment Measure-Revised measure of attachment; ECR-12, Experiences in Close Relationships-Short measure of attachment; DWM-S(A), Dysfunctional Working Models Scale (adapted) measure of problematic interpersonal beliefs. *p < 0.05, **p < 0.01.

### Associations between sociodemographic, clinical and relational factors, and therapy engagement and outcomes

We found no associations between gender, disability, long-term condition, ethnicity, presenting problem or psychosis risk (initial PQ-16) and sessions attended, not attended or cancelled. Age, depression (initial PHQ-9) and anxiety (initial GAD-7) were all negatively associated with sessions not attended. Depression (initial PHQ-9) was also negatively associated with sessions cancelled. Employment status was associated with sessions attended and not attended. See supplementary material (S3–S6) for all associations between sociodemographic and clinical factors and therapeutic engagement and outcomes.

Problematic interpersonal beliefs (DWM-S(A)) were positively associated with sessions attended but negatively associated with depression (PHQ-9) and anxiety (GAD-7) change scores, and psychosis risk (PQ-16). We found no associations between sociodemographic, clinical or relational factors and clinical outcomes, with the exception of age, which showed a moderate positive correlation with anxiety (GAD-7) change scores. See supplementary material (S7) for all associations between relational factors and therapeutic engagement and outcome.

## Discussion

This is the first study to assess feasibility, acceptability and signals of efficacy for CBT for depression and anxiety, minimally adapted for psychosis risk, in routine primary care services. The adaptations were developed in collaboration with people with direct experience of psychosis and family members, strengthening the quality and relevance of the study. Patient and public involvement colleagues emphasised the importance of asking about attenuated psychotic symptoms, and of non-judgement and warmth when asking about these experiences, given the social and internalised stigma associated with psychosis.

### Feasibility and acceptability of CBT for depression and anxiety adapted for psychosis risk

Rates of recruitment and completion of measures demonstrate feasibility, and rate of therapy completion demonstrates acceptability of the adapted therapy. Completion rates for measures and therapy favoured the adapted therapy. On average, people attended two more sessions, and cancelled one more session, in the CBT-PR group compared with best practice CBT, which may indicate some increased flexibility in practical arrangements in the adapted therapy group.

### Signals of efficacy

Routine measures of depression and anxiety signalled improved clinical and recovery outcomes for the adapted therapy. Given the evidence of causal links between insecure attachment and psychosis,^29–32^ as well as the widely recognised role of the therapeutic relationship in effecting change,^33^ signals of sustained change in relational measures are very encouraging. Moreover, signals of reduced psychosis risk are promising and somewhat surprising given the focus on depression and anxiety, rather than on psychotic symptoms. A definitive trial should monitor both psychosis risk and incidence of psychotic episodes over longer follow-up periods, and possible moderating effects of relational measures..

### Implications

Early detection and treatment of psychosis risk may yield considerable health and cost benefits.^34–37^ Early psychological interventions reduce depression, anxiety^1^ and transition to psychosis^8^ for people at risk of psychosis. This study is the first to demonstrate that minimally adapted CBT for depression and/or anxiety can be delivered in routine primary care services, and may close the gap in clinical outcomes for people with and without psychosis risk. By embedding the adapted therapy in established health care services, the intervention is highly scalable. A definitive trial will determine whether CBT adapted for psychosis risk improves outcomes for the affective presentations for which this group are seeking treatment. Longer-term follow-up data are needed to test whether effective treatments for depression and/or anxiety in turn have an impact on subsequent transition to psychosis.

### Limitations

This study is limited by the lack of randomisation to group. Additionally, as a feasibility study, we did not correct for multiple comparisons, increasing the risk of type I errors. Attrition of follow-up data limits conclusions drawn from 6- and 12-month measures. We completed no fidelity check or cost analyses. The therapists involved in the trial offered both standard and adapted CBT, though not all therapists in the service offered adapted CBT. While common in psychotherapy trials, having a self-selected group of therapists in the experimental arm may have biased outcomes. These limitations should be addressed in a definitive trial.

## Supporting information

Newman-Taylor et al. supplementary material Newman-Taylor et al. supplementary material

## Data Availability

The data that support the findings of this study are available from the corresponding author (K.N-.T.) upon reasonable request.

## Footnotes

a CBT led to a reduction in incidence of psychosis at 12 months (relative risk = 0.52, 95% CI 0.33–0.82) and at 18–48 months (relative risk = 0.60, 95% CI 0.42–0.84).^8^

b Previously Improving Access to Psychological Therapies (IAPT).

c Best practice CBT, defined as the recommended therapy protocols based on peer-reviewed evidence for treatment of depression and anxiety disorders^21^ is the basis for Talking Therapies provision.^14^

d Study dates: control period, 1 October 2021–31 March 2022 (CHR-P screening); recruitment for CBT-PR, 1 April 2022–31 March 2023; intervention and follow-up period, 1 April 2022–31 March 2024).

e BABCP is the UK accrediting body for CBT clinicians, supervisors and trainers.

f See preregistration for minor changes to planned analyses.

g In line with national guidelines, therapy is defined as two or more sessions;^38^ because this is arguably a low bar, we also report the numbers of sessions attended and cancelled.

## Supplementary material

The supplementary material can be found at https://doi.org/10.1192/bjo.2025.27

## Relevance statement

Practising psychiatrists will learn that people at high risk for psychosis may be identified in routine primary care settings, and that minimally adapted CBT for depression and anxiety is feasible, acceptable and likely to be beneficial for this population. This has implications for primary to tertiary pathways of care, and for the role of practising psychiatrists in shaping local services to ensure access to very early interventions, at a time when people are seeking help and from services equipped to offer evidence-based therapies. Further research is needed to determine whether these interventions affect subsequent rates of transition to psychosis.

## References

[1] Knight C , Russo D , Stochl J , Croudace T , Fowler D , Grey N , et al. Prevalence of and recovery from common mental disorder including psychotic experiences in the UK primary care improving access to psychological therapies (IAPT) programme. J Affect Disord 2020; 272: 84–90. 32379625 10.1016/j.jad.2020.04.015

[2] Newman-Taylor K , Maguire T , Smart T , Bayford E , Gosden E , Addyman G , et al. Screening for psychosis risk in primary mental health care services – implementation, prevalence and recovery trajectories. Br J Clin Psychol 2024; 63: 589–602. 10.1111/bjc.12490 38946546

[3] Perez J , Russo DA , Stochl J , Clarke J , Martin Z , Jassi C , et al. Common mental disorder including psychotic experiences: trailblazing a new recovery pathway within the Improving Access to Psychological Therapies programme in England. Early Interv Psychiatry 2018; 12: 497–504. 28509391 10.1111/eip.12434

[4] Yung AR , McGorry PD , McFarlane CA , Jackson HJ , Patton GC , Rakkar A. Monitoring and care of young people at incipient risk of psychosis. Schizophr Bull 1996; 22: 283–303. 8782287 10.1093/schbul/22.2.283

[5] Fusar-Poli P , Bonoldi I , Yung AR , Borgwardt S , Kempton MJ , Valmaggia L , et al. Predicting psychosis: meta-analysis of transition outcomes in individuals at high clinical risk. Arch Gen Psychiatry 2012; 69: 220–9. 22393215 10.1001/archgenpsychiatry.2011.1472

[6] Addington J , Cornblatt BA , Cadenhead KS , Cannon TD , McGlashan TH , Perkins DO , et al. At clinical high risk for psychosis: outcome for nonconverters. Am J Psychiatry 2011; 168: 800–5. 21498462 10.1176/appi.ajp.2011.10081191 PMC3150607

[7] Fusar-Poli P , Davies C , Solmi M , Brondino N , De Micheli A , Kotlicka-Antczak M , et al. Preventive treatments for psychosis: umbrella review (just the evidence). Front Psychiatry 2019; 10: 764. 31920732 10.3389/fpsyt.2019.00764 PMC6917652

[8] Mei C , van der Gaag M , Nelson B , Smit F , Yuen HP , Berger M , et al. Preventive interventions for individuals at ultra high risk for psychosis: an updated and extended meta-analysis. Clin Psychol Rev 2021; 86: 102005. 33810885 10.1016/j.cpr.2021.102005

[9] Ologundudu OM. Risk stratification for treatment decisions in people at ultra-high risk for psychosis: a cost-effectiveness analysis. MSc thesis The University of Western Ontario, 2020.

[10] DeVylder JE , Oh HY , Corcoran CM , Lukens EP. Treatment seeking and unmet need for care among persons reporting psychosis-like experiences. Psychiatr Serv 2014; 65: 774–80. 24534875 10.1176/appi.ps.201300254 PMC6483726

[11] Murphy J , Shevlin M , Houston J , Adamson G. A population based analysis of subclinical psychosis and help-seeking behavior. Schizophr Bull 2012; 38: 360–7. 20709763 10.1093/schbul/sbq092 PMC3283148

[12] Phalen PL , Rouhakhtar PR , Millman ZB , Thompson E , DeVylder J , Mittal V , et al. Validity of a two-item screen for early psychosis. Psychiatry Res 2018; 270: 861–8. 30551336 10.1016/j.psychres.2018.11.002 PMC6296822

[13] National Health Service (NHS) Mental Health Taskforce. *The NHS Five Year Forward View for Mental Health*. NHS Mental Health Taskforce, 2016 (https://www.england.nhs.uk/mental-health/taskforce/).

[14] Clark DM. Realizing the mass public benefit of evidence-based psychological therapies: the IAPT program. Annu Rev Clin Psychol 2018; 14: 159–83. 29350997 10.1146/annurev-clinpsy-050817-084833 PMC5942544

[15] Wakefield S , Kellett S , Simmonds-Buckley M , Stockton D , Bradbury A , Delgadillo J. Improving Access to Psychological Therapies (IAPT) in the United Kingdom: a systematic review and meta-analysis of 10-years of practice-based evidence. Br J Clin Psychol 2021; 60: 1–37. 32578231 10.1111/bjc.12259

[16] Hepgul N , King S , Amarasinghe M , Breen G , Grant N , Grey N , et al. Clinical characteristics of patients assessed within an Improving Access to Psychological Therapies (IAPT) service: results from a naturalistic cohort study (Predicting Outcome Following Psychological Therapy; PROMPT). BMC Psychiatry 2016; 16: 52. 26920578 10.1186/s12888-016-0736-6 PMC4769576

[17] Drake RJ , Husain N , Marshall M , Lewis SW , Tomenson B , Chaudhry IB , et al. Effect of delaying treatment of first-episode psychosis on symptoms and social outcomes: a longitudinal analysis and modelling study. Lancet Psychiatry 2020; 7: 602–10. 32563307 10.1016/S2215-0366(20)30147-4 PMC7606908

[18] Salazar de Pablo G , Guinart D , Armendariz A , Aymerich C , Catalan A , Alameda L , et al. Duration of untreated psychosis and outcomes in first-episode psychosis: systematic review and meta-analysis of early detection and intervention strategies. Schizophr Bull 2024: 50: 771–83. 38491933 10.1093/schbul/sbae017 PMC11283197

[19] Skivington K , Matthews L , Simpson SA , Craig P , Baird J , Blazeby JM , Boyd KA , et al. A new framework for developing and evaluating complex interventions: update of Medical Research Council guidance. BMJ 2021; 374: n2061. 34593508 10.1136/bmj.n2061 PMC8482308

[20] Eldridge SM , Lancaster GA , Campbell MJ , Thabane L , Hopewell S , Coleman CL , et al. Defining feasibility and pilot studies in preparation for randomised controlled trials: development of a conceptual framework. PLoS ONE 2016; 11: e0150205. 26978655 10.1371/journal.pone.0150205 PMC4792418

[21] Roth AD , Pilling S. *A Competence Framework for the Supervision of Psychological Therapies*. University College London, 2008 (www.ucl.ac.uk/CORE/).

[22] Ising HK , Veling W , Loewy RL , Rietveld MW , Rietdijk J , Dragt S , et al. The validity of the 16-item version of the Prodromal Questionnaire (PQ-16) to screen for ultra high risk of developing psychosis in the general help-seeking population. Schizophr Bull 2012; 38: 1288–96. 22516147 10.1093/schbul/sbs068 PMC3713086

[23] Kroenke K , Spitzer RL , Williams JB. The PHQ-9: validity of a brief depression severity measure. J Gen Intern Med 2001; 16: 606–13. 11556941 10.1046/j.1525-1497.2001.016009606.x PMC1495268

[24] Spitzer RL , Kroenke K , Williams JB , Löwe B. A brief measure for assessing generalized anxiety disorder: the GAD-7. Arch Intern Med 2006; 166: 1092–7. 16717171 10.1001/archinte.166.10.1092

[25] Mundt JC , Marks IM , Shear MK , Greist JM. The Work and Social Adjustment Scale: a simple measure of impairment in functioning. Br J Psychiatry 2002; 180: 461–4. 11983645 10.1192/bjp.180.5.461

[26] Pollard C , Bucci S , MacBeth A , Berry K. The revised psychosis attachment measure: measuring disorganized attachment. Br J Clin Psychol 2020; 59: e12249. 32415698 10.1111/bjc.12249 PMC7496745

[27] Lafontaine M-F , Brassard A , Lussier Y , Valois P , Shaver PR , Johnson SM. Selecting the best items for a short-form of the experiences in close relationships questionnaire. Eur J Psychol Assess 2016; 32: 140–54.

[28] Perris C , Fowler D , Skagerlind I , Olsson M , Thorsson C. Development and preliminary application of a new scale for assessing dysfunctional working models of self and others (DWM-S) in severely disturbed patients. Acta Psychiatr Scand 1998; 98: 219–23. 9761409 10.1111/j.1600-0447.1998.tb10070.x

[29] Newman-Taylor K , Sood M , Rowe AC , Carnelley KB. The impact of repeated attachment priming on paranoia, mood and help-seeking intentions in an analogue sample. Brain Sci 2021; 11: 1257. 34679322 10.3390/brainsci11101257 PMC8533775

[30] Sood M , Carnelley K , Newman-Taylor K. How does attachment imagery for paranoia work? Cognitive fusion and beliefs about self and others mediate the impact on paranoia and anxiety. Psychol Psychother Theory Res Pract 2021; 94: 973–93. 10.1111/papt.12354 34145722

[31] Sood M , Carnelley KB , Newman-Taylor K. Do emotion regulation strategies mediate the attachment–paranoia association? An experimental study of repeated attachment imagery priming and stress buffering. Psychol Psychother Theory Res Pract 2022; 95: 781–806. 10.1111/papt.12398 PMC9543866 35570714

[32] Sood M , Newman-Taylor K. Cognitive fusion mediates the impact of attachment imagery on paranoia and anxiety. Cogn Ther Res 2020; 44: 1150–61.

[33] Newman-Taylor K , Bentall R. Cognitive behavioural therapy for psychosis: the end of the line or time for a new approach? Psychol Psychother Theory Res Pract 2024; 97: 4–18. 10.1111/papt.12498 37804105

[34] Hegelstad WTV , Larsen TK , Auestad B , Evensen J , Haahr U , Joa I , et al. Long-term follow-up of the TIPS early detection in psychosis study: effects on 10-year outcome. Am J Psychiatry 2012; 169: 374–80. 22407080 10.1176/appi.ajp.2011.11030459

[35] Singh SP. Early intervention in psychosis. Br J Psychiatry 2010; 196: 343–5. 20435956 10.1192/bjp.bp.109.075804

[36] Stafford MR , Jackson H , Mayo-Wilson E , Morrison AP , Kendall T. Early interventions to prevent psychosis: systematic review and meta-analysis. BMJ 2013; 346: f185. 23335473 10.1136/bmj.f185 PMC3548617

[37] Williams R , Ostinelli EG , Agorinya J , Minichino A , De Crescenzo F , Maughan D , et al. Comparing interventions for early psychosis: a systematic review and component network meta-analysis. EClinicalMedicine 2024; 70: 102537. 38516103 10.1016/j.eclinm.2024.102537 PMC10955207

[38] National Health Service (NHS) England. *NHS Talking Therapies for Anxiety and Depression Manual 2018 [updated]*. NHS England, 2024 (https://www.england.nhs.uk/publication/the-improving-access-to-psychological-therapies-manual/).

